# Minds in movement: embodied cognition in the age of artificial intelligence

**DOI:** 10.1098/rstb.2023.0144

**Published:** 2024-10-07

**Authors:** Louise Barrett, Dietrich Stout

**Affiliations:** ^1^ Department of Psychology, University of Lethbridge, Lethbridge, Alberta T1K 3M4, Canada; ^2^ Department of Anthropology and Center for Mind, Brain, and Culture, Emory University, Atlanta, GA 30322, USA

**Keywords:** 4E cognition, LLM, embodiment, cognitive science, language and cognition, bio-behavioural synchrony

## Abstract

This theme issue brings together researchers from diverse fields to assess the current status and future prospects of embodied cognition in the age of generative artificial intelligence. In this introduction, we first clarify our view of embodiment as a potentially unifying concept in the study of cognition, characterizing this as a perspective that questions mind–body dualism and recognizes a profound continuity between sensorimotor action in the world and more abstract forms of cognition. We then consider how this unifying concept is developed and elaborated by the other contributions to this issue, identifying the following two key themes: (i) the role of language in cognition and its entanglement with the body and (ii) bodily mechanisms of interpersonal perception and alignment across the domains of social affiliation, teaching and learning. On balance, we consider that embodied approaches to the study of cognition, culture and evolution remain promising, but will require greater integration across disciplines to fully realize their potential. We conclude by suggesting that researchers will need to be ready and able to meet the various methodological, theoretical and practical challenges this will entail and remain open to encountering markedly different viewpoints about how and why embodiment matters.

This article is the part of this theme issue ‘Minds in movement: embodied cognition in the age of artificial intelligence’.

## 1. Introduction

Over the past quarter century and more, disciplines from anthropology to robotics have argued that cognition is concretely grounded in bodily sensation and movement. Starting in the 1990s, the concept of ‘embodied cognition’ was hailed by many as a major paradigm shift for cognitive science, promising new insight into everything from visual perception to child development, social cognition and language [1–3]. However, it is not clear to all that this promise has been realized. Embodied perspectives have been critiqued as more rhetorical than substantive [4], with core premises that are either ‘unacceptably vague’ or ‘nothing new’ [5]. Such concerns have only been amplified by the recent success of completely *dis*-embodied deep learning models in producing human-like linguistic and perceptual behaviour. Recently, this even led to the widely reported suggestion that Google’s Language Model for Dialogue Applications may already have achieved artificial sentience. The time is thus ripe for a critical reassessment of the role of the body in human cognition and experience.

This theme issue arises from an interdisciplinary workshop organized by the Center for Mind, Brain, and Culture (cmbc.emory.edu) in May 2023. Workshop participants are joined here by an array of international experts to assess the current status and future prospects of embodied approaches to cognition, culture and evolution. To this end, we approach embodied cognition not as a core body of theory to be tested but as a research programme that may (or may not) generate interesting and productive questions [6]. To assess this productivity, we have assembled contributors across disciplines ranging from anthropology to architecture, education, movement science, neuroscience, philosophy, primatology and psychology. In order to provide a balanced view, these contributions include not only laboratory experiments [7–9] and theoretical reviews [10–14] but also ethnographic case studies [15,16] and practical applications [17–19]. Educators in particular have been keen to apply embodied approaches to their practice. One recent review identified 247 articles on embodied teaching and learning in higher education, including 66 addressing teacher education [20]. Other areas of potential application include robotics [21], rehabilitation medicine [22], human–computer interaction [23] and architecture [24]. Within academia, embodied perspectives have been developed in fields ranging from sociocultural anthropology [25], Palaeolithic archaeology [26] and evolutionary neuroscience [27] to literature, theatre and art [28].

## 2. The concept of embodiment

Given the scope of this issue, it is important to further specify (or at least constrain) the unifying concept of ‘embodiment’. In the context of motor control, embodiment may mean little more than the common-sense observation that bodies constrain action [29]. For cognitive psychologists and philosophers, on the other hand, it might constitute a fundamental claim about the nature of the human mind [1]. Here we use the term in an inclusive way to refer to perspectives that question the duality of mind and body and view concrete perception and action in the world as critical to, and perhaps inseparable from, more ‘abstract’ forms of cognition. Embodiment in this sense has a deep history in the phenomenology of philosophers like Husserl [30] and Merleau-Ponty [31], the functional psychology of William James and John Dewey (e.g. [32]), anthropological theory stressing the importance of bodily techniques and habitual practices in the (re)production of cultural meaning [33,34], ecological psychology concepts of organism–environment coupling and active perception [35,36] and cognitive linguistic attempts to derive abstract semantics from concrete metaphors [37].

Contemporary interest in embodied cognition, including its more recent elaboration as ‘4E’ (embodied, embedded, enactive and extended) cognition [38], originated as a reaction to the perceived excesses of the cognitive revolution [1]. According to standard accounts, the behaviourist orthodoxy of the early twentieth century viewed ‘mind’ as a mystical concept unsuitable for scientific study owing to its inherent unobservability [39]. The emergence of information theory [40] and computer science [41] began to change this, providing a model of concretely observable information processing that helped validate internal cognitive processes as a topic of study [42]. Building on this computer analogy, theoreticians like Lashley [43], Miller [44] and Chomsky [45] initiated what later became known as the cognitive revolution. This work established linguistic syntax as a model for understanding human cognition as a hierarchically structured, rule-based, computational system of symbol manipulation. Importantly, this analogy posits an arbitrary relationship between form and meaning [46], leading to a vision of cognition as the purely internal manipulation of amodal symbols.

It is worth noting here, however, that this account of the decline and fall of behaviourism is overly simplistic, collapsing widely contrasting flavours of behaviourism into a monolith (i.e. Watson’s methodological behaviourism, Tolman’s purposive behaviourism, Hull’s drive theory and Skinner’s radical behaviourism) and misrepresenting radical behaviourism in particular [47,48]. Far from stating that mind should be excluded from scientific study owing to its unobservability, Skinner expressly denied the mind–body dualism inherent in such a position; after all, to exclude a private, internal mind is to implicitly accept that it exists [49]. Instead, like the pragmatists before him and Dewey especially, Skinner simply did not distinguish between an inner subjective world and an outer objective one [50]. For Skinner, the goals and reasons that a person or other animal might have for expressing a particular behaviour were seen as tangled up in the actions taken in the world and thus as components of the behaviour itself: ‘What is felt or introspectively observed is not some nonphysical world of consciousness, mind, or mental life but the observer’s own body’ [51, p. 18], and ‘cognitive psychologists like to say “the mind is what the brain does” but surely the body plays a part? The mind is what the body does. It is what the person does. In other words, it is behaviour’ [52, p. 784]. This is worth pointing out because, as noted by Barrett [47], such statements are almost indistinguishable from those of prominent 4E theorists, such as Andy Clark: ‘To thus take body and world seriously is to invite an emergentist perspective on many key phenomena—to see adaptive success as inhering as much in the complex interactions among body, world and brain as in the inner processes bound by skin and skull’ [53, p. 84]. The rise of 4E approaches can be seen, then, not just as a reaction to the excesses of the cognitive revolution but also as an attempt to restore the valuable pragmatist insights of the behaviourist programme.

Thus, while the computational approach continues to generate a huge body of productive research—recently including the application of deep learning algorithms for generative artificial intelligence (AI)—it has also drawn criticism on a number of points [1,2], some of which reflect Skinner’s earlier objections to ‘mentalism’. These include (i) the difficulty of relating amodal symbols back to perception and action (the symbol grounding problem), (ii) scepticism that human brains instantiate anything like the ‘trans-situationally’ invariant symbols posited by classical computational approaches, (iii) the assumed dichotomy between representations and processes (symbols versus rules), and (iv) the fundamentally Cartesian assumption that cognition is independent of, and can be studied without respect to, the physical substrates that instantiate it.

We can also add here the views of another of the original architects of the cognitive revolution, Jerry Bruner, who ended up profoundly disillusioned with what he saw as the ‘new reductionism’: ‘…what I and my friends thought the revolution was about in the late 1950s…[was] an all-out effort to establish meaning as the central concept of psychology’ [54, p. 2], but that ‘very early on…emphasis began shifting from “meaning” to “information”, from the *construction* of meaning to the *processing* of information. These are profoundly different matters’ ([54, p. 4], emphasis original). For Bruner, ‘the new cognitive science, the child of the revolution, has gained its technical successes at the price of dehumanizing the very concept of mind it had sought to reestablish in psychology, and it has thereby estranged much of psychology from the other human sciences and the humanities’ [54, p. 1].

There is ample room for disagreement over whether these various critiques call for another revolutionary paradigm shift or more of a friendly amendment to mainstream cognitive science [4]. For example, the increasingly influential active inference (cf. predictive processing, Bayesian brain, predictive coding) theory of brain function [55], though commonly associated with embodied and enactive perspectives [56], is equally amenable to more classically computational/representational interpretations [57,58]. In this issue, Dove [10] argues that recent research with large language models (LLMs) supports a less dichotomous view and that some aspects of human cognition are best understood by combining embodied and computational approaches. From a more applied perspective, Di Paolo *et al*. [17] argue that generative AI could be deployed as a ‘cognitive prosthesis’ to better structure the active learning of school children.

The impacts of early computer science and subsequent reactions can be traced in other fields as well. According to D’Andrade [59], the demise of behaviourism and ascendance of the computer metaphor of mind led to a similar shift in sociocultural anthropology from an emphasis on overt cultural ‘habits’ (i.e. behaviour patterns) to a view of culture as internalized knowledge and rules for action, essentially describing a cultural ‘programme’ (e.g. [60]) or even a symbolic ‘information-holding system with functions similar to that of cellular DNA’ [59, p. 198]. However, this informational conception of culture quickly waned in influence. For example, the symbolic anthropologist Clifford Geertz located meaning within the minds of individuals but also stressed the enactive nature of culture, arguing that ‘it is through the flow of behaviour—or, more precisely, social action—that cultural forms find articulation’ [61, p. 17]. Such enactive culture extends beyond the transmission of information to the concrete instantiation of social institutions as repeated patterns of activity, structured relations between people and constructed environments. For example, Giddens [62] described a dialectical process of ‘structuration’ in which the actions of individual agents simultaneously create and are constrained by emergent social structures. Bourdieu [63] similarly underlined the role of embodied practice in reproducing culture, using the term ‘habitus’ to describe non-discursive dispositions of thought and action that emerge from repeated participation in structured activity. This perspective is mechanistically elaborated in the ‘ethnokinesiology’ of Ting *et al*. [12] and applied by Noel *et al*. [18] to inform the architectural design of the spaces that structure human activity. Provocative parallels may be drawn between such social theories and the diachronic organism–environment coupling and self-structuring information flows of 4E cognition (e.g. [56]). Downey [15] and Saraei *et al*. [16] develop this link by exploring the physiological, perceptual and affective dimensions of patterned cultural practices in real-world athletic (freedivers) and religious contexts respectively. Downey [15] suggests that this embodied perspective may provide a path to better integrate biology into cultural research. It is thus important to recognize that biology, and particularly the grand unifying biological theory of evolution, has also had a mixed relationship with embodiment.

Modern evolutionary theory has its origins in the mid-twentieth-century synthesis of Darwinian selection with the emerging field of population genetics. By the time the physical structure and replication mechanisms of DNA were established in the 1950s, the influence of computer science and information theory made a computational interpretation of this genetic ‘code’ seem almost self-evident (e.g. [64]). The influential ‘selfish gene’ concept advanced by Dawkins [65] took this to a logical extreme, reducing organisms to the status of vehicles for replicating genes that were the informational units in an algorithmic process of selection. This extreme view has, however, drawn extensive criticism. Particularly prominent was Ernst Mayr [66], who argued that selection acts directly on whole organisms and only indirectly affects genes. Others now advocate a more catholic multi-level selection framework [67]. This fundamental issue regarding the units of selection has broad implications that have not always been emphasized in mainstream evolutionary theory. Understanding the evolution of organisms and phenotypes (cf. ‘bodies’) is a very different prospect from modelling changes in gene frequencies. Phenotypic evolution may involve a wider array of causal pathways, such as non-genetic inheritance, niche construction, somatic constraint, developmental plasticity and behaviour-led evolution to name just a few. Such extra-genetic mechanisms have recently been integrated as an ‘extended evolutionary synthesis’ [68], although once again there is room for disagreement over their novelty and impact [69]. Nevertheless, this phenotypic, body-oriented perspective clearly seeks to undo the classical [70] dichotomy between proximate ‘how’ (e.g. somatic and developmental mechanisms) and ultimate ‘why’ (i.e. history of genetic selection) causes in evolutionary biology.

Thus, there is an interesting convergence across ‘embodied’ perspectives on cognition, culture and evolution, all of which seek to move away from simplifying software/hardware, mind/body, organism/environment and cause/mechanism dichotomies of the mid-twentieth-century information-processing paradigm. The result is a more integrated approach to cognition as emerging from dynamic interactions across behavioural, developmental, historical and evolutionary timescales (e.g. [71,72]). Fragaszy *et al*. [11] provide a detailed working example of such multi-level interaction at a relatively fine-grained spatiotemporal scale in their multi-fractal cascade analysis of suprapostural dexterity, tool use and the sense of agency. At a broader conceptual level, ‘extended’ multi-level approaches have become increasingly prevalent across research on human evolution [73,74], evolutionary neuroscience [27,75,76], comparative [77,78] and developmental [29,79] psychology. Notable applications include biologically inspired AI and robotics [80,81]. With respect to human evolution, Fragaszy *et al*. [11] apply their multi-fractal cascade approach to assess the importance of bipedalism and associated postural changes to the emergence of distinctive human tool use capacities, thus bringing bodies firmly into a field that more typically focuses on the brain and cognition (e.g. [82,83]). Relatedly, de Vignemont & Farnè [13] discuss the evolution and adaptive functions of peri-personal space [84], a unique perceptual zone flexibly defined by current bodily abilities (e.g. reach) and thought to be important for both tool use and bodily self-awareness in primates. At a broader level, Van Woerkum & Barrett [14] critique the mainstream comparative psychology approaches that seek to reveal ‘deep’ cognitive similarities by dismissing ‘superficial’ differences in perception, action and context. They argue that this practice actively fabricates similarity and cloaks difference—a process they term ‘anthropofabrication’. Grandchamp des Raux *et al*. [9] implement these perspectives experimentally, with results that suggest that the emergence of putatively ‘abstract’ common-sense reasoning in children is grounded in bodily interactions with the environment.

## 3. Key themes

At least two key themes emerge in the consideration of embodied cognition across the diverse contributions to this theme issue: the role of language in cognition and of the body in self/other awareness. The former theme has historically been a core explanatory target for embodiment theory (and its critics) and is of obvious relevance to assessing the implications of recent advances in generative AI LLMs. The latter theme addresses mechanisms of interpersonal perception, prediction and alignment relevant to everything from consciousness to social affiliation, teaching and learning.

### (a) Language and cognition

As Dove [10] explains, the ‘origin story’ of embodied cognition is very closely tied to the so-called symbol grounding problem [85]: the difficulty of relating abstract symbols back to the concrete concepts and percepts to which they refer. Embodied cognition proposes to solve this problem by grounding meaning in modal simulations of perceptual–motor sensation [2]. This, however, leads to the symmetrical problem of explaining how abstract and apparently disembodied concepts like ‘justice’ can be constructed from such concrete experiential simulations, which Dove refers to as the symbol *ungrounding* problem. One response is to argue that even apparently abstract concepts are more concretely grounded than we might assume (e.g. [86]), particularly if we take into account affective and situational simulations [2]. However, Dove and many others (e.g. [87]) would instead advocate some version of a dual code [88] theory, in which embodied simulation is supplemented by learned symbolic systems such as language and number. In the framework developed by Deacon [89], symbolic meaning is grounded because it is constructed from percepts/simulations that achieve reference through similarity (iconicity). These are linked to tokens (e.g. words) through associative/statistical learning (indexicality), thus allowing the construction of a complex system of reference and association between these tokens (symbolism). Seccia & Goldin-Meadow’s discussion of the role of gesture in mathematical learning provides a concrete example of the continued relevance of iconic and indexical reference to the learning and real-world human use of formal symbol systems [19].

From this perspective, language and number may be seen as cognitive prostheses [17,90] providing ‘access to an external symbol system that has different computational properties from non-linguistic grounded cognition’ [10]. Such a perspective thus echoes earlier work by the Russian psychologist, Lev Vygotsky, who argued that all higher mental functions are mediated by ‘psychological tools’, such as language, signs and symbols [91]. Most recently, Heyes [92, p. 1] has referred to such symbol systems as ‘cognitive gadgets’ that ‘enable our minds to go further, faster, and in different directions than the minds of any other animal’. Van Woerkum & Barrett [14] follow Noë [93] in describing this intimate relationship between human language and cognition as an irreducible ‘entanglement’. With respect to memory, for example, they argue that oral storytelling, mnemonic techniques, literacy, visual art and more ‘have now become so fused together with whatever biological capacities we possess, that this… now just *is* the human form of memory’ [14].

This entanglement is nicely illustrated in the ethnographic example of freediving presented by Downey [15]. Although freediving skill is quite literally embodied in changes to heart rate and peripheral vasoconstriction responses that can only be acquired through physical training and experience, these ‘patterned practices’ are themselves products of a linguistically mediated community of practice. For example, a key part of training is the discursive knowledge that elevated CO_2_ levels bring on involuntary diaphragm contractions that, while unpleasant, are neither damaging nor dangerous in themselves; this communally acquired knowledge allows divers to cognitively re-appraise their experience, regulate their affective response and persist well past the critical ‘struggle phase’ that marks an upper limit for naive divers. As Downey [15] explains ‘An expert dive response is a combined biological and cultural achievement … [that] involves volitional self-regulation of emotions, bodily priming through patterned behaviour, community practical and scientific learning … Expert divers and the community of practice study their bodies’ reactions and their performances reflexively. They seek to influence their circulatory systems even though they have incomplete volitional control or even perception, recognizing factors that trigger or disrupt bradycardia and vasoconstriction, including mental and emotional activity’. In this example, the external symbol system provided by language acts as a tool for communicating information between individuals, but also as a tool of perception (e.g. through labelling, abstraction and associations between symbols), introspection and metacognitive evaluation, cognitive reappraisal of affect and self-regulation [10,90,94]. Indeed, the centrality of language in the cultural reproduction of knowledge and skills has led some to speculate that demands for teaching and learning may have been a key selective pressure driving the evolution of language [95–97].

Current hypotheses of language evolution generally recognize the centrality of embodied production and perception, including both speech and gesture [98,99]. As Ting *et al*. [12] describe, speech is one of the most complex movement systems in nature and must be learned through extensive experimentation in a physically and socially structured environment that provides appropriate ecological consequences. Insofar as the development of external symbol systems like language requires the production and perception of increasing numbers of discriminable physical tokens (utterances and gestures) [98], the emergence of symbolically mediated dual code cognition is itself dependent on evolving capacities for embodied perception and action. Furthermore, as elaborated by several contributors to this issue (Li *et al*. [7], Payne & Catmur [8], Saraei *et al*. [16]), such capacities may also be important to establishing the inter-individual neurobehavioural alignment thought to support the social affiliation and shared meaning [100,101] critical to language use and many other cooperative human activities. Such observations led Stout [72,102] to propose a ‘perceptual–motor hypothesis’ that grounds human cognitive evolution in the elaboration of phylogenetically ancient primate perceptual–motor systems for body awareness and engagement with the world. While the dual code cognition of humans appears to be unlike that of any other extant species, van Woerkum & Barrett [14] point out that all animals employ situated sensory–motor strategies to ‘do’ cognition. In this sense, external symbol systems are just another unique form of situated cognitive scaffolding.

This view of language as a biologically and culturally evolved tool for thinking that is thoroughly entangled with non-linguistic grounded cognition may help to resolve a deceptive dichotomy and to explain the peculiar LLM pattern of ‘surprising successes and disappointing failures’ [10]. In a recent review, Mahowald *et al*. [103] argue that LLMs excel at reproducing the rules and statistical regularities of formal language competence (i.e. systematic symbol manipulation) but fall short on what they call functional language competence, including reasoning, world knowledge, situation modelling and social understanding. They suggest [103, p. 533] that addressing this shortcoming will require a modular organization ‘mimicking the division of labour between formal and functional competence in the human brain’. Although these authors clearly envision such integration as entirely computational/representational, an alternative view is that deficits in functional language use correspond fairly directly to the limitations of ungrounded symbol manipulation and that human-like integration would entail human-like entanglement. If anything, this would make LLMs more, rather than less, interesting for the study of human cognition. As Dove concludes, ‘Reframing LLMs as an exaggeration of an important aspect of human cognition enables a kind of rapprochement between the two sides of the debate… With respect to cognitive science, LLMs can be viewed as biologically implausible models of the way language-based experience might help unground a fundamentally embodied conceptual system. With respect to AI, the need for grounding provides an impetus to explore larger systems that include action- and perception-oriented representations’.

### (b) Self and other in alignment

As outlined above, internal simulation is key to many theories of embodied cognition [104]. Indeed, embodied simulation using forward (i.e. predictive) models of action (e.g. [105]) has been proposed as a unifying framework for cognitive science [1]. Such suggestions are not without critics; however, as such models are often explicitly computational and advocates of ‘radical’ embodied cognition (e.g. Fragaszy *et al*. [11,78]), would eschew the use of any such computational metaphors for cognitive processes. As reviewed by Allen & Friston [57], there is a range of views on the utility of describing internal neural dynamics as mapping to, modelling, simulating or representing the body and world. Clark and colleagues [56,58] have attempted to resolve this tension using a predictive processing/active inference framework derived from the free-energy principle of Friston [55]. This formalism derives from thermodynamics rather than computer science, but its application to cognition nevertheless involves a translation to information–theoretical (i.e. representational) units. The multi-fractal cascade approach of Fragaszy *et al*. [11] offers another interesting direction to pursue.

Leaving aside the issue of how prediction/expectation might actually be implemented in organism–environment systems, it is apparent that anticipation is a central feature of behaviour and cognition. As reviewed in several contributions (Fragaszy *et al*. [11]; Li *et al*. [7]; Payne & Catmur [8]; de Vignemont & Farnè [13]) comparison between expected and experienced perceptual consequences of action is thought to play a critical role in the sense of agency [106], self–other discrimination and social cognition [107]. The flip side of using sensorimotor prediction to discriminate self from other is its potential role in coordinating action and the potential for alignment between self and other to promote affiliation by eroding this sense of difference. Ting *et al*. [12] develop the concept of a ‘motor accent’ arising from a misalignment of motor concept topologies when an agent attempts to use their existing movement patterns in a new context. Such accents are a perceived phenomenon that only exists on the observer’s end, and Ting *et al*. [12] suggest they may play a role in maintaining group identity. More broadly, interactive synchrony across multiple levels of physiological and neural activity has been proposed to support implicit mentalizing, empathy, communication, learning and social affiliation [100,108,109]. Interestingly, this list substantially overlaps with the ‘disappointing failures’ of ungrounded LLMs discussed in the previous section.

Li *et al.* [7] present an experimental investigation of interbrain synchronization [101] during cooperative learning [110]. Such synchronization has been found to correlate with shared meaning during linguistic communication and with enhanced learning outcomes in experimental and real-world classroom settings. However, the mechanisms producing such synchronization, and consequently, its status as cause versus symptom of effective communication, have remained unclear. In their study of collaborative poetry analysis, Li *et al*. [7] find that spontaneous, synchronized body movements contribute to interbrain synchronization, which in turn facilitates learning. In conjunction with Seccia & Goldin-Meadow’s [19] insights into the role of gesture in math learning, this brings us closer to understanding and appreciating the central role of the body even in abstract, symbol-based reasoning and communication. Interestingly, Li *et al*. [7] found this effect only in participant dyads with relatively homogenous expertise (either high or low) and not in mixed dyads. This suggests an important role for shared frames of reference and/or enhanced social affiliation with (perceived to be) similar others in effective collaboration and learning. It is thus interesting to note that synchronized movement can also be a powerful mechanism for promoting such social affiliation in the first place, with potentially useful pedagogical implications.

These results are also consistent with longstanding speculation regarding the role of synchronized communal activities in the evolution of human societies, reviewed here by Saraei *et al*. [16]. In their field study of the Islamic ritual *Salat al Jama’ah*, these authors attempt to distinguish between different candidate mechanisms for group-level physiological synchronization: following a single leader, flock-like synchronization with immediate neighbours, or attuning directly to the structure of the ritual. Using wearable devices to record body posture, autonomic responses and spatial proximity, they find that worshippers synchronize their movements with their nearest neighbours, whereas physiological alignment is primarily driven by the religious leader. These dual process results point to the complex ways in which ritual activity can drive interpersonal alignment and the social connections it fosters.

Of course, human self-awareness involves more than just a sense of agency and body ownership. Payne & Catmur [8] distinguish between this idea of a ‘bodily self’ and a more abstract ‘conceptual self’ including personality attributes, attitudes, beliefs, preferences, social roles and material possessions. They probe this distinction through an experimental manipulation of the ‘Enfacement’ illusion in which a participant has their face stroked while watching another person’s face being stroked synchronously. Similar to the famous ‘rubber hand illusion’ [111], this is sufficient to cause the participant to experience some degree of ‘ownership’ over the other’s face as indicated by questionnaire and perceptual judgements tasks, as well as broader effects on affective ratings and judgements about attractiveness, trustworthiness, personality similarity and implicit attitudes. Payne & Catmur [8] find that this illusion is enhanced by greater initial conceptual similarity (operationalized as gender independent of physical similarity), suggesting a complex relationship between the construction of conceptual and bodily selves.

Such complexity echoes more general debates about the nature of human consciousness, which has been characterized as everything from a concretely embodied feeling [112] to a form of computational self-reference [113]. In fact, different people likely mean many different things when they talk about consciousness. Damasio [112], for example, distinguishes between the protoself (a neural ‘mapping’ of the physical state of the body), a ‘core’ consciousness roughly comparable to the bodily self and an ‘extended’ consciousness that includes autobiographical narratives comparable to one’s ‘identity’ or conceptual self. It is not clear whether a purely symbolic narrative consciousness that was not built on top of embodied sensation would be possible or even conceptually coherent. It will thus be very interesting to investigate what kind of consciousness, if any, ungrounded LLMs might potentially instantiate and how it would differ from the inherently embodied human experience.

## 4. Conclusion: the continued relevance of embodied cognition

On the one hand, readers will not be surprised to learn that we believe contributions to this issue make a strong case for the continued relevance and utility of embodied perspectives on cognition, culture and evolution. On the other hand, it is clear that realizing this promise will present important methodological, theoretical and pragmatic challenges, not least of which is the need for communication and collaboration across traditionally distinct disciplines with very different approaches, terminology and assumptions, even with respect to what ‘embodiment’ means. What seems clear is that bodily sensation and movement are complex, variable, context-sensitive and deeply intertwined with cognition, emotion and culture. Progress will require the development of new methods for the rich recording and analysis of human behaviour in real-world contexts combined with a deeper appreciation of the complex interactions between bio-behavioural mechanisms, social dynamics and cultural systems of meaning. Relevant processes range in scale from neurons to societies and call for an approach that is both inclusive and integrative, embracing quantitative, qualitative and applied research, focused experiments as well as long-term observations and detailed case studies as well as broad comparative work.

## Data Availability

This article has no additional data.

## References

[1] Glenberg AM , Witt JK , Metcalfe J . 2013 From the revolution to embodiment: 25 years of cognitive psychology. Perspect. Psychol. Sci. **8** , 573–585. (10.1177/1745691613498098) 26173215

[2] Barsalou LW . 2008 Grounded cognition. Annu. Rev. Psychol. **59** , 617–645. (10.1146/annurev.psych.59.103006.093639) 17705682

[3] Smith LB , Thelen E . 1994 A dynamic systems approach to the development of cognition and action. Cambridge, MA: MIT Press/Bradford Books. (10.7551/mitpress/2524.001.0001)

[4] Carney J . 2020 Thinking avant la lettre: a review of 4E cognition. Evol. Stud. Imaginative Cult. **4** , 77–90. (10.26613/esic/4.1.172) 32457930 PMC7250653

[5] Goldinger SD , Papesh MH , Barnhart AS , Hansen WA , Hout MC . 2016 The poverty of embodied cognition. Psychon. Bull. Rev. **23** , 959–978. (10.3758/s13423-015-0860-1) 27282990 PMC4975666

[6] Wołoszyn K , Hohol M . 2017 Commentary: the poverty of embodied cognition. Front. Psychol. **8** , 845. (10.3389/fpsyg.2017.00845) 28588543 PMC5440554

[7] Li Y , Su C , Pan Y . 2024 Spontaneous movement synchrony as an exogenous source for interbrain synchronization in cooperative learning. Phil. Trans. R. Soc. B **379** , 20230155. (10.1098/rstb.2023.0155) 39155721 PMC11391278

[8] Payne B , Catmur C . 2024 Embodiment in the enfacement illusion is mediated by self–other overlap. Phil. Trans. R. Soc. B **379** , 20230146. (10.1098/rstb..2023.0146) 39155718 PMC11391314

[9] Grandchamp des Raux H , Ghilardi T , Soderberg C , Ossmy O . 2024 The role of action concepts in physical common sense: insights from late childhood. Phil. Trans. R. Soc. B **379** , 20230154. (10.1098/rstb.2023.0154) 39155719 PMC11391279

[10] Dove G . 2024 Symbol ungrounding: what the successes (and failures) of large language models reveal about human cognition. Phil. Trans. R. Soc. B **379** , 20230149. (10.1098/rstb.2023.0149) 39155725 PMC11529626

[11] Fragaszy D , Kelty-Stephen D , Mangalam M . 2024 How bipedalism shapes humans’ actions with hand tools. Phil. Trans. R. Soc. B **379** , 20230152. (10.1098/rstb.2023.0152) 39155723 PMC11391300

[12] Ting L , Gick B , Kesar T , Xu J . 2024 Ethnokinesiology: towards a neuromechanical understanding of cultural differences in movement. Phil. Trans. R. Soc. B **379** , 20230485. (10.1098/rstb.2023.0485) 39155720 PMC11529631

[13] de Vignemont F , Farnè A . 2024 Peripersonal space: why so last-second? Phil. Trans. R. Soc. B **379** , 20230159. (10.1098/rstb.2023.0159) 39155714 PMC11529623

[14] van Woerkum B , Barrett L . 2024 Anthropofabrication and the redressing of memory: an embodied approach to comparative cognition. Phil. Trans. R. Soc. B **379** , 20230145. (10.1098/rstb.2023.0145) 39155716 PMC11529621

[15] Downey G . 2024 Skill building in free diving as an example of embodied culture. Phil. Trans. R. Soc. B **379** , 20230150. (10.1098/rstb.2023.0150) 39155712 PMC11391316

[16] Saraei M , Paxton A , Xygalatas D . 2024 Aligned bodies, united hearts: embodied emotional dynamics of an Islamic ritual. Phil. Trans. R. Soc. B **379** , 20230162. (10.1098/rstb.2023.0162) 39155713 PMC11391295

[17] Di Paolo LD , White B , Guénin-Carlut A , Constant A , Clark A . 2024 Active inference goes to school: the importance of active learning in the age of large language models. Phil. Trans. R. Soc. B **379** , 20230148. (10.1098/rstb.2023.0148) 39155715 PMC11391319

[18] Noel V , Parikh S , Wilson J , Grunert S . 2024 A mindful body approach to creating architecture: movement, models and spatial experiences. Phil. Trans. R. Soc. B **379** , 20230153. (10.1098/rstb.2023.0153) 39155724 PMC11391302

[19] Seccia A , Goldin-Meadow S . 2024 Gesture can help children learn math: how researchers can work with teachers to make gesture studies applicable to classrooms. Phil. Trans. R. Soc. B **379** , 20230156. (10.1098/rstb.2023.0156) 39155717 PMC11391277

[20] Hegna HM , Ørbæk T . 2024 Traces of embodied teaching and learning: a review of empirical studies in higher education. Teach. Higher. Educ. **29** , 420–441. (10.1080/13562517.2021.1989582)

[21] Pigozzi F , Woodman S , Medvet E , Kramer-Bottiglio R , Bongard J . 2023 Morphology choice affects the evolution of affordance detection in robots (ed. J Bongard ). In Proceedings of the Genetic and Evolutionary Computation Conference (GECCO ’23), July 2023, Lisbon, Portugal, pp. 211–219. New York, NY: Association for Computing Machinery. (10.1145/3583131.3590505)

[22] Verschure PFMJ , Páscoa Dos Santos F , Sharma V . 2023 Redefining stroke rehabilitation: mobilizing the embodied goal-oriented brain. Curr. Opin. Neurobiol. **83** , 102807. (10.1016/j.conb.2023.102807) 37980804

[23] Pustejovsky J , Krishnaswamy N . 2021 Embodied human computer interaction. Künstl. Intell. **35** , 307–327. (10.1007/s13218-021-00727-5)

[24] Arbib MA . 2021 When brains meet buildings. New York, NY: Oxford University Press. (10.1093/med/9780190060954.001.0001). See https://academic.oup.com/book/35996.

[25] Ingold T . 2021 The perception of the environment: essays on livelihood, dwelling and skill. London, UK: Routledge. (10.4324/9781003196662)

[26] Wynn T , Overmann KA , Malafouris L . 2021 4E cognition in the Lower Palaeolithic. Adapt. Behav. **29** , 99–106. (10.1177/1059712320967184)

[27] Cisek P , Hayden BY . 2022 Neuroscience needs evolution. Phil. Trans. R. Soc. B **377** , 20200518. (10.1098/rstb.2020.0518) 34957841 PMC8710875

[28] Cook A . 2018 4E cognition and the humanities. In The Oxford handbook of 4E cognition (eds A Newen , L De , S Gallagher ), pp. 875–890. New York, NY: Oxford University Press. (10.1093/oxfordhb/9780198735410.013.47)

[29] Adolph KE , Hoch JE . 2019 Motor development: embodied, embedded, enculturated, and enabling. Annu. Rev. Psychol. **70** , 141–164. (10.1146/annurev-psych-010418-102836) 30256718 PMC6320716

[30] Husserl E . 2012 Cartesian meditations: an introduction to Phenomenology. In Introduction to Phenomenology. Springer Science & Business Media.

[31] Merleau-Ponty M . 1962 Phenomenology of perception. New York, NY: Humanities Press.

[32] Dewey J . 1896 The reflex arc concept in psychology. Psychol. Rev. **3** , 357–370. (10.1037/h0070405)

[33] Mauss M . 1936 Les techniques Du Corps. J. de psychol. **32** , 271–293.

[34] Bourdieu P . 1977 Outline of a theory of practice. Cambridge, UK: Cambridge University Press. (10.1017/CBO9780511812507)

[35] Gibson JJ . 1966 The senses considered as perceptual systems. Boston, MA: Houghton Mifflin.

[36] Reed ES . 1996 Encountering the world: toward an ecological psychology. New York, NY: Oxford University Press.

[37] Lakoff G , Johnson M . 1980 Metaphors we live by. Chicago, IL: University of Chicago Press.

[38] Newen A , De L , Gallagher S . 2018 The Oxford handbook of 4E cognition. New York, NY: Oxford University Press.

[39] Miller GA . 2003 The cognitive revolution: a historical perspective. Trends Cogn. Sci. **7** , 141–144. (10.1016/S1364-6613(03)00029-9) 12639696

[40] Shannon CE , Weaver W . 1949 The mathematical theory of communication. Urbana, IL: University of Illinois Press.

[41] Turing AM . 1950 Computing machinery and intelligence. Mind **LIX** , 433–460. (10.1093/mind/LIX.236.433)

[42] Crowther-Heyck H . 1999 Miller, language, and the computer metaphor and mind. Hist. Psychol. **2** , 37–64. (10.1037//1093-4510.2.1.37) 11623619

[43] Lashley K . 1951 The problem of serial order in behavior. In Cerebral mechanisms in behavior (ed. LA Jeffress ), pp. 112–136. New York, NY: John Wiley.

[44] Miller GA , Pribram KH , Galanter E . 1960 Plans and the structure of behavior. New York, NY: Holt, Reinhart and Winston. (10.1037/10039-000)

[45] Chomsky N . 1957 *Syntactic structures* . The Hague, The Netherlands: Mouton. (10.1515/9783112316009)

[46] de Saussure F . 1966 Course in general linguistics. New York, NY: McGraw-Hill.

[47] Barrett L . 2012 Why Behaviorism isn’t Satanism. In The Oxford handbook of comparative evolutionary psychology (eds TK Shackelford , J Vonk ), pp. 17–38. Oxford, UK: Oxford University Press. (10.1093/oxfordhb/9780199738182.001.0001)

[48] Malone JC . 2009 Psychology: Pythagoras to present. Cambridge, MA: MIT Press.

[49] Skinner BF . 1984 The operational analysis of psychological terms. Behav. Brain Sci. **7** , 547–553. (10.1017/S0140525X00027187)

[50] Baum WM . 1994 Understanding Behaviorism: science, behavior, and culture. New York, NY: Harper Collins College Publishers.

[51] Skinner BF . 1974 About Behaviorism, 1st edn. New York, NY: Knopf [distributed by Random House].

[52] Skinner BF . 1987 Whatever happened to psychology as the science of behavior? Am. Psychol. **42** , 780–786. (10.1037/0003-066X.42.8.780)

[53] Clark A . 1997 Being there: putting brain, body, and world together again. Cambridge, MA: MIT Press. (10.7551/mitpress/1552.001.0001)

[54] Bruner J . 1990 Acts of meaning. Cambridge, MA: Harvard University Press.

[55] Friston K . 2010 The free-energy principle: a unified brain theory? Nat. Rev. Neurosci. **11** , 127–138. (10.1038/nrn2787) 20068583

[56] Clark A . 2013 Whatever next? Predictive brains, situated agents, and the future of cognitive science. Behav. Brain Sci. **36** , 181–204. (10.1017/S0140525X12000477) 23663408

[57] Allen M , Friston KJ . 2018 From cognitivism to autopoiesis: towards a computational framework for the embodied mind. Synthese **195** , 2459–2482. (10.1007/s11229-016-1288-5) 29887647 PMC5972168

[58] Constant A , Clark A , Friston KJ . 2020 Representation wars: enacting an armistice through active inference. Front. Psychol. **11** , 598733. (10.3389/fpsyg.2020.598733) 33488462 PMC7817850

[59] D’Andrade RG . 1982 Cultural meaning systems. In Behavioral and social science research: a national resource (eds RM Adams , NJ Smelser , DJ Treiman ), pp. 197–236. National Academies Press.

[60] Goodenough WH . 1957 Cultural anthropology and linguistics. In Report on the seventh Anuual round table meeting on linguistics and language study (ed. P Garvin ). Washington DC: Georgetown University. (Georgetown University Mongraph Series, Language and Linguistics).

[61] Geertz C . 1973 The interpretation of cultures: selected essays. New York, NY: Basic Books.

[62] Giddens A . 1976 New rules of sociological method: a positive critique of interpretative Sociologies. New York, NY: Basic Books.

[63] Bourdieu P . 1972 Outline of a theory of practice. Cambridge, UK: Cambridge University Press.

[64] Smith JM . 2000 The concept of information in biology. Philos. Sci. **67** , 177–194. (10.1086/392768)

[65] Dawkins R . 1976 The selfish gene. New York, NY: Oxford University Press.

[66] Mayr E . 1997 The objects of selection. Proc. Natl Acad. Sci. USA **94** , 2091–2094. (10.1073/pnas.94.6.2091) 9122151 PMC33654

[67] Wilson DS , Madhavan G , Gelfand MJ , Hayes SC , Atkins PWB , Colwell RR . 2023 Multilevel cultural evolution: from new theory to practical applications. Proc. Natl Acad. Sci. **120** , e2218222120. (10.1073/pnas.2218222120) 37036975 PMC10120078

[68] Laland KN , Uller T , Feldman MW , Sterelny K , Müller GB , Moczek A , Jablonka E , Odling-Smee J . 2015 The extended evolutionary synthesis: its structure, assumptions and predictions. Proc. R. Soc. B **282** , 20151019. (10.1098/rspb.2015.1019) PMC4632619 26246559

[69] Laland K *et al* . 2014 Does evolutionary theory need a rethink? Nature **514** , 161–164. (10.1038/514161a) 25297418

[70] Mayr E . 1961 Cause and effect in biology. Science **134** , 1501–1506. (10.1126/science.134.3489.1501) 14471768

[71] Veissière SPL , Constant A , Ramstead MJD , Friston KJ , Kirmayer LJ . 2020 Thinking through other minds: a variational approach to cognition and culture. Behav. Brain Sci. **43** , e90. (10.1017/S0140525X19001213) 31142395

[72] Stout D . 2021 The cognitive science of technology. Trends Cogn. Sci. **25** , 964–977. (10.1016/j.tics.2021.07.005) 34362661

[73] Fuentes A . 2016 The extended evolutionary synthesis, ethnography, and the human niche: toward an integrated anthropology. Curr. Anthropol. **57** , S13–S26. (10.1086/685684)

[74] Stock J , Will M , Wells JC . 2023 Niche construction, plasticity, and inclusive inheritance: rethinking human origins with the extended evolutionary synthesis, part 1. Paleoanthropol. 205–233. (10.48738/2023.iss2.123)

[75] Hecht EE , Barton SA , Rogers Flattery CN , Meza Meza A . 2023 The evolutionary neuroscience of domestication. Trends Cogn. Sci. (Regul. Ed.) **27** , 553–567. (10.1016/j.tics.2023.03.008) 37087363

[76] Dennis EJ , El Hady A , Michaiel A , Clemens A , Tervo DRG , Voigts J , Datta SR . 2021 Systems neuroscience of natural behaviors in rodents. J. Neurosci. Nat. Behav. Rodent. **41** , 911–919. (10.1523/JNEUROSCI.1877-20.2020) PMC7880287 33443081

[77] Leavens DA , Bard KA , Hopkins WD . 2019 The mismeasure of ape social cognition. Anim. Cogn. **22** , 487–504. (10.1007/s10071-017-1119-1) 28779278 PMC6647540

[78] Barrett L . 2011 Beyond the brain: how body and environment shape animal and human minds. Princeton, NJ: Princeton University Press. (10.1515/9781400838349)

[79] Byrge L , Sporns O , Smith LB . 2014 Developmental process emerges from extended brain–body–behavior networks. Trends Cogn. Sci. **18** , 395–403. (10.1016/j.tics.2014.04.010) 24862251 PMC4112155

[80] Kudithipudi D *et al* . 2022 Biological underpinnings for lifelong learning machines. Nat. Mach. Intell. **4** , 196–210. (10.1038/s42256-022-00452-0)

[81] Blackiston D , Kriegman S , Bongard J , Levin M . 2023 Biological robots: perspectives on an emerging interdisciplinary field. Soft Robot. **10** , 674–686. (10.1089/soro.2022.0142) 37083430 PMC10442684

[82] Hecht EE , Pargeter J , Khreisheh N , Stout D . 2023 Neuroplasticity enables bio-cultural feedback in Paleolithic stone-tool making. Sci. Rep. **13** , 2877. (10.1038/s41598-023-29994-y) 36807588 PMC9938911

[83] Osiurak F , Reynaud E . 2020 The elephant in the room: what matters cognitively in cumulative technological culture. Behav. Brain Sci. **43** , 1–66. (10.1017/S0140525X19003236) 31739823

[84] Noel JP , Bertoni T , Serino A . 2021 Peri-personal space as an interface for self-environment interaction: a critical evaluation and look ahead. In The world at our fingertips: a Multidisciplinary exploration of Peripersonal space (eds F de Vignemont , A Serino , HY Wong , A Farnè ), pp. 17–46. (10.1093/oso/9780198851738.001.0001)

[85] Harnad S . 1990 The symbol grounding problem. Physica D. Nonlinear Phenomena. **42** , 335–346. (10.1016/0167-2789(90)90087-6)

[86] Lakoff G , Johnson M . 1980 The metaphorical structure of the human conceptual system. Cogn. Sci. **4** , 195–208. (10.1207/s15516709cog0402_4)

[87] Barsalou LW . 2010 Grounded cognition: past, present, and future. Top. Cogn. Sci. **2** , 716–724. (10.1111/j.1756-8765.2010.01115.x) 25164052

[88] Paivio A . 1971 Imagery and verbal processes. New York, NY: Holt, Rinehart and Winston.

[89] Deacon TW . 1997 The symbolic species: the co-evolution of language and the brain. New York, NY: W.W. Norton.

[90] Clark A . 2006 Language, embodiment, and the cognitive niche. Trends Cogn. Sci. **10** , 370–374. (10.1016/j.tics.2006.06.012) 16843701

[91] Vygotsky L . 1962 Thought and language. Cambridge, MA: M.I.T. Press. (10.1037/11193-000). See http://content.apa.org/books/11193-000.

[92] Heyes C . Cognitive gadgets: the cultural evolution of thinking. Cambridge, MA: Harvard University Press. (10.4159/9780674985155). See https://www.degruyter.com/document/doi/10.4159/9780674985155/html.

[93] Noë A . 2023 The entanglement: how art and philosophy make us what we are. Princeton, NJ: Princeton University Press.

[94] Shea N , Boldt A , Bang D , Yeung N , Heyes C , Frith CD . 2014 Supra-personal cognitive control and metacognition. Trends Cogn. Sci. (Regul. Ed.) **18** , 186–193. (10.1016/j.tics.2014.01.006) PMC3989995 24582436

[95] Stout D , Chaminade T . 2012 Stone tools, language and the brain in human evolution. Phil. Trans. R. Soc. B **367** , 75–87. (10.1098/rstb.2011.0099) 22106428 PMC3223784

[96] Laland KN . 2017 The origins of language in teaching. Psychon. Bull. Rev. **24** , 225–231. (10.3758/s13423-016-1077-7) 27368625 PMC5325857

[97] Stout D . 2018 Archaeology and the evolutionary neuroscience of language: the technological pedagogy hypothesis. Interact. Stud. **19** , 256–271. (10.1075/is.17033.sto)

[98] Arbib MA . 2012 How the brain got language: the mirror system hypothesis. Oxford, UK: Oxford University Press. (10.1093/acprof:osobl/9780199896684.001.0001). See https://academic.oup.com/book/32785.

[99] Levinson SC , Holler J . 2014 The origin of human multi-modal communication. Phil. Trans. R. Soc. B **369** , 20130302. (10.1098/rstb.2013.0302) 25092670 PMC4123681

[100] Shamay-Tsoory SG , Saporta N , Marton-Alper IZ , Gvirts HZ . 2019 Herding brains: a core neural mechanism for social alignment. Trends Cogn. Sci. **23** , 174–186. (10.1016/j.tics.2019.01.002) 30679099

[101] Hasson U , Ghazanfar AA , Galantucci B , Garrod S , Keysers C . 2012 Brain-to-brain coupling: a mechanism for creating and sharing a social world. Trends Cogn. Sci. **16** , 114–121. (10.1016/j.tics.2011.12.007) 22221820 PMC3269540

[102] Stout D , Hecht EE . 2017 Evolutionary neuroscience of cumulative culture. Proc. Natl Acad. Sci. USA **114** , 7861–7868. (10.1073/pnas.1620738114) 28739892 PMC5544267

[103] Mahowald K , Ivanova AA , Blank IA , Kanwisher N , Tenenbaum JB , Fedorenko E . 2024 Dissociating language and thought in large language models. Trends Cogn. Sci. **28** , 517–540. (10.1016/j.tics.2024.01.011) 38508911 PMC11416727

[104] Barsalou LW . 1999 Perceptual symbol systems. Behav. Brain Sci. **22** , 577–609; (10.1017/s0140525x99002149) 11301525

[105] Wolpert DM , Doya K , Kawato M . 2003 A unifying computational framework for motor control and social interaction. Phil. Trans. R. Soc. Lond. B **358** , 593–602. (10.1098/rstb.2002.1238) 12689384 PMC1693134

[106] Haggard P . 2017 Sense of agency in the human brain. Nat. Rev. Neurosci. **18** , 196–207. (10.1038/nrn.2017.14) 28251993

[107] Guzman M , Bird G , Banissy MJ , Catmur C . 2016 Self–other control processes in social cognition: from imitation to empathy. Phil. Trans. R. Soc. B **371** , 20150079. (10.1098/rstb.2015.0079) 26644597 PMC4685524

[108] Hasson U , Frith CD . 2016 Mirroring and beyond: coupled dynamics as a generalized framework for modelling social interactions. Phil. Trans. R. Soc. B. **371** , 20150366. (10.1098/rstb.2015.0366) 27069044 PMC4843605

[109] Feldman R . 2017 The Neurobiology of human attachments. Trends Cogn. Sci. 80–99. (10.1016/j.tics.2016.11.007) 28041836

[110] Pan Y , Cheng X , Hu Y . 2023 Three heads are better than one: cooperative learning brains wire together when a consensus is reached. Cereb. Cortex **33** , 1155–1169. (10.1093/cercor/bhac127) 35348653

[111] Botvinick M , Cohen J . 1998 Rubber hands “feel” touch that eyes see. Nature **391** , 756. (10.1038/35784) 9486643

[112] Damasio A . 1999 The feeling of what happens: body and emotion in the making of consciousness. New York, NY: Harcourt Brace & Company.

[113] Hofstadter D . 1979 Gödel, escher, bach: an eternal golden braid. New York, NY: Basic Books.

